# Supplementary material for: Quality of hospital labour and delivery care: A multilevel analysis in Southern Nations and Nationalities People’s Region of Ethiopia
Source: PLoS One. 2024 Jun 18;19(6):e0285058. doi: 10.1371/journal.pone.0285058 (PMC11185448; doi:10.1371/journal.pone.0285058)
Supplement: S2 Checklist — (DOCX) [file pone.0285058.s002.docx]

**S3 STROBE-checklist-v4-combined-PlosMedicine**

STROBE Statement—Checklist of items that should be included in reports of ***cross-sectional studies***

|  | Item No | Recommendation | Page  No. | Relevant text from manuscript |
| --- | --- | --- | --- | --- |
| **Title and abstract** | 1 | (*a*) Indicate the study’s design with a commonly used term in the title or the abstract | 2 | Facility-based cross-sectional study; Multilevel analysis |
|  |  | (*b*) Provide in the abstract an informative and balanced summary of what was done and what was found | 2 | Evaluated the quality of routine L&D care provided by government hospitals and identified patient-and hospital-level factors |
|  |  |  | 3 | On average, the hospitals met two-thirds of the standards for L&D care quality (SD 10.9 percentage points). The quality of L&D care score was significantly higher for women who had a history of any danger sign (β = 5.66) and for women who were cared for at a teaching hospital (β = 12.1). Hospitals with lower volume and more resources available for L&D care also had higher L&D quality scores. |
| Introduction | | |  |  |
| Background/rationale | 2 | Explain the scientific background and rationale for the investigation being reported | 4 | Ethiopia is not on track to meet SDGs of reducing MMR to less than 70 per 100,000 live births and ending preventable deaths of newborns to at least as low as 12 per 1,000 live births by 2030. |
|  |  |  | 5 | The contemporary level of quality of hospital-based L&D care as well as the patient- and hospital-level factors associated with higher L&D care quality in SSA in general and in Ethiopia, specifically, remain largely unknown. |
| Objectives | 3 | State specific objectives, including any prespecified hypotheses | 5 | To describe the quality of hospital-based L&D care as measured by adherence to L&D quality of care standards set forth by the Ethiopian Ministry of Health and to identify patient- and hospital-factors associated with higher quality care. |
| Methods | | |  |  |
| Study design | 4 | Present key elements of study design early in the paper |  |  |
| Setting | 5 | Describe the setting, locations, and relevant dates, including periods of recruitment, exposure, follow-up, and data collection | 6 | Government hospitals (n=20) in the Ethiopian region of SNNPR from Nov 11 to Dec 10, 2016. |
| Participants | 6 | (*a*) Give the eligibility criteria, and the sources and methods of selection of participants | 6 | Births were excluded (n=16) if the birthing woman had any high-risk factors (e.g., pre-eclampsia or previous scar), if the neonate was not alive at birth, or if the birthing woman developed complications during the during the first hour following the birth. |
| Variables | 7 | Clearly define all outcomes, exposures, predictors, potential confounders, and effect modifiers. Give diagnostic criteria, if applicable | 7-8 | Dependent and independent variables described. |
| Data sources/ measurement | 8* | For each variable of interest, give sources of data and details of methods of assessment (measurement). Describe comparability of assessment methods if there is more than one group | 7-8 | The outcome was calculated for each birth as the percentage of all relevant standards fulfilled, with possible values from 0%-100%. The percentage fulfilled was reported for the relevant standards overall as well as within 7 domains.  Patient-level independent variables included the woman’s age, number of previous births, number of skilled attendants involved in care process, presence of any danger sign in current pregnancy.  Hospital-level independent variables included teaching hospital status, mean number of births in the hospital in the previous year, number of fulltime skilled attendants in the L&D ward on the assessment day, whether the hospital had offered refresher training on L&D care in the previous 12 months, and the extent of resources available (measured on a 0-100% scale) to provide quality L&D care. |
| Bias | 9 | Describe any efforts to address potential sources of bias | 6 | Direct observation might have influenced measures of performance due to the inevitable Hawthorne effect. We sought to minimize this bias by observing hospital staff for a full month and assured anonymity in all data reports. |
| Study size | 10 | Explain how the study size was arrived at | 6 | We undertook a non-participant observation study conducted with all normal vaginal deliveries of women 18 years and older occurring in government hospitals (n=20) of SNNPR from Nov 11 to Dec 10, 2016. |
| Quantitative variables | 11 | Explain how quantitative variables were handled in the analyses. If applicable, describe which groupings were chosen and why | 7-8 | The independent variables were grouped as Level 1 (woman level) and Level 2 (hospital level) with the aim of adjusting for the potential clustering effect, which was tested through the multilevel modelling. |
| Statistical methods | 12 | (*a*) Describe all statistical methods, including those used to control for confounding | 7-8 | Descriptive analysis, centering of all continuous independent variables on the grand means, t-test, and multilevel modelling. |
|  |  | (*b*) Describe any methods used to examine subgroups and interactions | 9 | Interaction terms were created, and their effects tested. |
|  |  | (*c*) Explain how missing data were addressed | 9 | Cases with missing data for key variables were excluded from further analysis. |
|  |  | (*d*) If applicable, describe analytical methods taking account of sampling strategy | 8-9 | Descriptive analysis and mixed effects multilevel linear regression modelling were performed. Intra-class correlation coefficients (ICCs) were calculated. |
|  |  | (*e*) Describe any sensitivity analyses |  | N/A due to non-considerable missingness in the dataset. |
| Results | | |  |  |
| Participants | 13* | (a) Report numbers of individuals at each stage of study—eg numbers potentially eligible, examined for eligibility, confirmed eligible, included in the study, completing follow-up, and analysed | 9 | A total of 1,351 labouring mothers were approached and gave consent to participate in the study; 16 were later excluded due to non-normal progression of labour resulting in an analytic sample of 1,335 women (98.8% of those approached for the study). |
|  |  | (b) Give reasons for non-participation at each stage | 9 | 16 were later excluded due to non-normal progression of labour. |
|  |  | (c) Consider use of a flow diagram |  | N/A because, the analytic sample of 1,335 women was used throughout. |
| Descriptive data | 14* | (a) Give characteristics of study participants (eg demographic, clinical, social) and information on exposures and potential confounders | 9-11 | Characteristics of women and hospitals |
|  |  | (b) Indicate number of participants with missing data for each variable of interest | 9-10 | Table 1 – age, number of previous pregnancies, number of previous births, etc. |
| Outcome data | 15* | Report numbers of outcome events or summary measures | 12 | Summary score for quality of L&D care |
| Main results | 16 | (*a*) Give unadjusted estimates and, if applicable, confounder-adjusted estimates and their precision (eg, 95% confidence interval). Make clear which confounders were adjusted for and why they were included | 17-18 | Factors associated with quality of L&D care process |
|  |  | (*b*) Report category boundaries when continuous variables were categorized |  | NA because such categorization was not used for further analysis, |
|  |  | (*c*) If relevant, consider translating estimates of relative risk into absolute risk for a meaningful time period |  | NA |
| Other analyses | 17 | Report other analyses done—eg analyses of subgroups and interactions, and sensitivity analyses |  | NA |
| Discussion | | |  |  |
| Key results | 18 | Summarise key results with reference to study objectives | 20 | We found that on average hospitals met only two-thirds of the standards for quality of L&D care, with particularly poor performance in three domains of care: initial assessment of the woman in labour, AMTSL, and interpersonal communication. |
| Limitations | 19 | Discuss limitations of the study, taking into account sources of potential bias or imprecision. Discuss both direction and magnitude of any potential bias | 22 | The findings should be interpreted in light of several limitations. |
| Interpretation | 20 | Give a cautious overall interpretation of results considering objectives, limitations, multiplicity of analyses, results from similar studies, and other relevant evidence | 22 | The findings should be interpreted in light of several limitations. |
| Generalisability | 21 | Discuss the generalisability (external validity) of the study results | 22 | The sample was restricted to hospitals from SNNPR; results may differ in other geographies. |
| Other information | | |  |  |
| Funding | 22 | Give the source of funding and the role of the funders for the present study and, if applicable, for the original study on which the present article is based |  | N/A. Due to the restriction on the manuscript submission portal to include funding information within the manuscript, the below statement was added directly to the portal instead.  “Funding was provided to NBB by NIH Fogarty International Center under the Global Health Equity Scholars Program Grant #R25 TW009338 (<https://www.fic.nih.gov/>). The funder had no role in study design, data collection and analysis, decision to publish, or preparation of the manuscript. |

*Give information separately for exposed and unexposed groups.

**Note:** An Explanation and Elaboration article discusses each checklist item and gives methodological background and published examples of transparent reporting. The STROBE checklist is best used in conjunction with this article (freely available on the Web sites of PLoS Medicine at <http://www.plosmedicine.org/>, Annals of Internal Medicine at <http://www.annals.org/>, and Epidemiology at <http://www.epidem.com/>). Information on the STROBE Initiative is available at [www.strobe-statement.org](http://www.strobe-statement.org).
