# Supplementary material for: Quality of hospital labour and delivery care: A multilevel analysis in Southern Nations and Nationalities People’s Region of Ethiopia
Source: PLoS One. 2024 Jun 18;19(6):e0285058. doi: 10.1371/journal.pone.0285058 (PMC11185448; doi:10.1371/journal.pone.0285058)
Supplement: S1 Appendix — (PDF) [file pone.0285058.s003.pdf]

S1 Appendix

Domain specific mean percentage scores of routine L&D care process by hospital in SNNPR, Ethiopia, 2016

Name of hospital	Mean percentage scores by domain of care process															
	Initial assessment of the woman		Care during 2nd and 3rd stages of labour		Intrapartum interpersonal communication		Partograph use		AMTSL tasks		Immediate and essential newborn care		IP practices		Overall mean score	
	Mean	SD	Mean	SD	Mean	SD	Mean	SD	Mean	SD	Mean	SD	Mean	SD	Mean	SD
Adare	53.3	12.3	55.3	13.5	32.8	33.2	66.7	18.3	45.4	10.2	80.4	5.7	41.3	11.8	53.6	6.9
Arba Minch	64.6	13.6	71.8	18	59.8	24.9	71.9	34.7	39.5	12.5	91	9.9	77.5	9.7	68	8.5
Bona	41	9.6	75.6	10.8	71.2	30.7	89.4	10. 9	42.2	11.9	88.2	13.4	66.1	5.8	67.7	5
Bonga	60.2	13.2	66.1	11	3.7	13.2	62.2	24.4	40.8	12.3	93.3	15.7	76.3	8.6	57.4	5.4
Butajira	68.6	12.4	64.5	12	50.4	4.5	52.1	19.4	40.7	12.2	87.3	5.7	70.2	14.8	62.1	4.9
Chencha	67	13.9	90.7	11.4	51.7	34	95.4	7.6	37.9	14.3	93.5	6.3	90.5	7.9	75.8	6.3
Dilla	72.9	12.6	75.4	18.5	28.4	25.1	68.9	21.9	42.2	11.9	88.9	6.7	64.6	9	62.3	8.9
Durame	43.2	9.8	62.8	10.1	46.4	13	69.8	10.6	43.3	11.4	84.3	7.6	76.6	10.6	60.2	4.5
Gidole	59.6	15.6	76.7	22.9	40.2	41.7	85.5	28.9	37.2	12.6	98.9	4.3	89.8	10.3	70.8	9.4
Halaba	65.2	12.4	54.3	22.7	82.9	32.8	93.9	16.1	34	14.7	70.7	18.7	70.4	14.8	68.5	11.2
Hawassa Referral	47.7	14.3	56.5	18.4	76.8	29.1	74.9	16.4	24.5	10.9	87.2	9.9	78.7	6.1	63.8	7.7
Hossana	60.6	10	81.8	16.8	12.2	21.6	84.1	4.9	45.8	9. 8	87	5.3	73.8	8.8	64.8	4.5
Jinka	61.6	10.3	96.1	11.3	42.3	33.4	79.5	18.6	47.4	8.3	90	12.4	77.1	5.4	70.5	7
Karat	30.6	15.1	54	21.2	95	15.8	63.3	29.2	50.5	0.2	85.6	11.8	76.3	7.1	65	6.5
Leku	54.4	7.8	75.7	10.4	70.7	40.3	67.9	10.8	50.5	0	91.3	6.8	79.6	7.8	69.9	6.8
Mizan Aman	74.3	9.2	75.8	22.6	41.1	43	94.1	11.9	47.4	8.4	85.7	11.3	57.8	11.4	67.9	10
Sawula	74.8	12.7	99.1	4.1	97.7	10.7	99.6	2.5	50.5	0	99.3	2.8	87.8	1.8	87.4	2
Tercha	49.4	11.7	96.9	9.3	14.6	34.5	86.1	19.5	46. 7	9.1	74.6	31.8	73.8	9.6	64.8	9.5
Wolaita Sodo	48	13.7	60.6	18.4	36.1	38.1	63.9	24.6	45.2	10.2	81.2	11.9	55.9	21.3	56.6	9.6
Yirgalem	72	12	96.9	7.3	100	0	100	0	45.2	10.3	85.3	8.8	87.7	1.5	85.2	2.2
**Overall mean score**	59.6	15.6	72.9	20.6	47.2	38.4	76.3	23.9	42.4	12.2	86.8	11.8	71.8	16.6	**66.6**	**10.9**
